# Household air pollution and the lung microbiome of healthy adults in Malawi: a cross-sectional study

**DOI:** 10.1186/s12866-016-0803-7

**Published:** 2016-08-11

**Authors:** Jamie Rylance, Anstead Kankwatira, David E. Nelson, Evelyn Toh, Richard B. Day, Huaiying Lin, Xiang Gao, Qunfeng Dong, Erica Sodergren, George M. Weinstock, Robert S Heyderman, Homer L. Twigg, Stephen B. Gordon

**Affiliations:** 1 Department of Clinical Sciences, Liverpool School of Tropical Medicine, Liverpool, L3 5QA UK; 2 Department of Medicine, Indiana University, Indianapolis, IN USA; 3 Malawi-Liverpool-Wellcome Trust Clinical Research Programme, Blantyre, Malawi; 4 Department of Microbiology and Immunology, Indiana University School of Medicine, Indianapolis, IN USA; 5 Center for Biomedical Informatics, Department of Public Health Sciences, Loyola University Chicago, Maywood, IL 60153 USA; 6 The Jackson Laboratory for Genomic Medicine, Farmington, Connecticut 06032 USA

**Keywords:** Respiratory microbiome, Household air pollution, Alveolar macrophage, Petrobacter

## Abstract

**Background:**

Domestic combustion of biomass fuels, such as wood, charcoal, crop residue and dung causes Household Air Pollution (HAP). These inhaled particulates affect more than half of the world’s population, causing respiratory problems such as infection and inflammatory lung disease. We examined whether the presence of black carbon in alveolar macrophages was associated with alterations in the lung microbiome in a Malawi population.

**Methods:**

Bronchoalveolar lavage samples from 44 healthy adults were sequenced using 16S rDNA amplification to assess microbial diversity, richness and relative taxa abundance. Individuals were classified as high or low particulate exposure as determined by questionnaire and the percentage of black carbon within their alveolar macrophages.

**Results:**

Subjects in the low and high particulate groups did not differ in terms of source of fuels used for cooking or lighting. There was no difference in alpha or beta diversity by particulate group. Neisseria and Streptococcus were significantly more abundant in samples from high particulate exposed individuals, and Tropheryma was found less abundant. Petrobacter abundance was higher in people using biomass fuel for household cooking and lighting, compared with exclusive use of electricity.

**Conclusions:**

Healthy adults in Malawi exposed to higher levels of particulates have higher abundances of potentially pathogenic bacteria (Streptococcus, Neisseria) within their lung microbiome. Domestic biomass fuel use was associated with an uncommon environmental bacterium (Petrobacter) associated with oil-rich niches.

## Background

Globally, most inhaled particulate matter derives from the domestic combustion of biomass fuels such as wood, charcoal, crop residue and dung [1, 2]. This Household Air Pollution (HAP) is associated with 4.3 million deaths per year from respiratory disease, including 900,000 childhood deaths from pneumonia [3–6]. Alterations in microbial populations in the lung caused by particle exposure could explain increased rates of respiratory infection in subjects exposed to HAP. Inhaled particulates are known to drive inflammation in the lung, to alter microbial binding to respiratory epithelium, and to act as vehicles delivering microbial molecules to the distal airways.

In healthy lungs, previously thought to be sterile environments, communities of bacteria, together with fungi and viruses form the microbiome. Variations in this microbiome may either reflect or drive mucosal inflammation and immune function [7]. Extensive sequencing of bacterial 16S rDNA from the lungs of healthy individuals has revealed the common presence of phyla such as Proteobacteria, Bacteroidetes, Actinobacteria and Firmicutes [8].

Study of the alterations in lung microbiota resulting from environmental exposures is a nascent field with the potential to explain the pathophysiological mechanisms of lung disease. Cigarette smoking does not appear to be associated with significant changes in the lung microbiome in a US cohort [9]. However, the effects of exposure to other environmental sources of particulates, particularly in low income countries, have not yet been described.

We hypothesised that, in healthy people, HAP would be associated with alterations in the lung microbiome in terms of diversity, richness and the relative abundance of various microbial taxa. Furthermore, prior comparative analysis of geographical differences in microbiota prevalence identified Petrobacter in more than a third of a group sampled in Malawi, but none in a US cohort. Petrobacter is a gram negative, aerobic bacterium identified in 2004 from oil reservoir samples. Given this niche, we hypothesised that the prevalence in a Malawi group would be due to inhalation of smoke from biomass fuel use: we present evidence of this association in this paper [10, 11].

## Methods

### Participants and bronchoscopy

Healthy, non-smoking, HIV-negative adults aged 18 to 50 were recruited from peri-urban communities in Blantyre, Malawi from May 2009 to December 2012. Ethical approval was granted by the College of Medicine REC, University of Malawi and Liverpool School of Tropical Medicine REC (P.03/10/916 and 09.69 respectively), and written consent was obtained. Bronchoalveolar lavage (BAL) fluid was obtained as previously described [12], filtered through gauze and centrifuged immediately at 400 g for 10 min. The cell pellet was processed as described below. Acellular supernatants were stored at -80 °C for batch extraction and sequencing. Structured interviews determined participants’ demographics and type of fuel used for heating, cooking and lighting. The stated “main source” of each was used as a classifier in analyses.

### Quantification of particulate within cells, and participant selection

The BAL cell pellet was re-suspended in RPMI 1640, and cells were counted using a Neubauer chamber. Cytospin preparations of macrophages (Thermo Shandon, UK) were imaged at 40x by light microscopy. Fifty fields from each experiment were analysed using freely available digital image analysis software (Image SXM, www.ImageSXM.org.uk), as previously described [13]. The samples for this study were drawn from a larger bronchoscopic study of 128 volunteers, all of whom had particulate imaging and quantification. Samples were sequentially identified for microbiome analysis from the highest and lowest particulate of available and adequate BAL samples. No further stratification or selection strategy was used.

### Microbiome analysis

DNA was extracted from BAL supernatants using DNAse/RNase free reagents and materials and a DNeasy kit (Qiagen, CA, USA). Ribosomal 16S subunit rDNA sequencing was performed at the Genome Institute (Washington University, MO, USA) as previously described [9]. Briefly, 27 F-534R primers for the hypervariable regions 1 to 3 (V1V3) were utilized. The 16 s rDNA sequencing was performed on the Roche 454 FLX Titanium platform and processed with the Mothur package v1.29 [14] based on its standard operative procedure (http://www.mothur.org/wiki/454_SOP). Briefly, sequence reads were demultiplexed into individual samples based on perfect match to the barcode sequences. Primers and barcodes were trimmed from each read and low-quality and chimeric sequences were removed with default Mothur parameters with one minor adjustment: the trump symbol was not included at *filter.seqs()* step due to our observation that its resulting in over-removal of aligned reads. The remaining high-quality 16S sequences (420 ± 15.9 bp) from each sample were classified using the RDP Classifier v2.5 with the default threshold value of 0.8 from phylum to genus level [15].

### Data analysis

Comparison of alveolar macrophage black carbon content with subject demographics: Two-way contingency tables were created using high/low alveolar macrophage black carbon content as one category and subject demographics as the other category. Forty-four demographics features include sex, cook fuel, cook location, heat, smoking, light fuel, and living conditions were examined. Fisher’s exact test was applied for the analysis [16].

Microbiome Analysis: To account for the uneven sequencing depth of each sample, all 44 samples were normalized using subsampling without replacement at depth 843 reads, and the subsampling was repeated for 10 times. The averaged read count among the 10 permutations was used in subsequent analysis. Alpha diversity richness was measured using Observed taxa number, Chao 1 and ACE indices, and diversity evenness was assessed using Shannon, Simpson’s (1-D), and Pielou indices [17]. These were compared between particulate groups using Wilcoxon Rank Sum tests. Difference of alpha diversity between groups was analysed by linear model with and without confounding factors, such as age, gender, cooking location, type of cook fuel and light fuel. Beta-diversity was visualized by non-metric multidimensional scaling using Bray-Curtis dissimilarity [18, 19] by the R *ecodist* package [20]. The PERMANOVA test in the R *vegan* package was used to test whether high biomass and low biomass cohorts form distinct clusters based on Bray-Curtis dissimilarities among the samples [21]. Multivariate dispersion of groups was compared using the *betadisper()* command in R *vegan* package to test for homogeneity of variance in high biomass and low biomass cohorts [22].

Differences in the abundance of specific genera between groups was analysed using negative binomial (NB) models and adjusted for differences in age, gender, cooking location, and smoking status prior to 6 months before the study (all participants were non-smokers for the 6 months immediately prior). To filter extremely low abundant taxa in our analysis, we limited this analysis to bacteria that were present in greater than 1 % abundance in at least one cohort.

## Results

### Participants

**Fig. 1:**
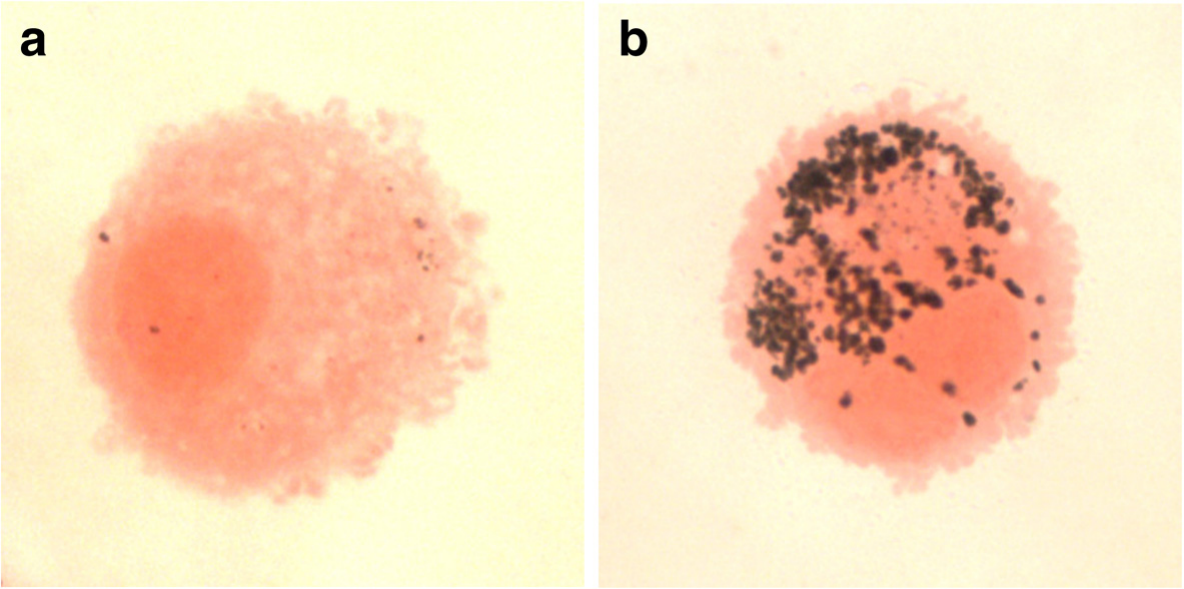
Representative images of macrophage staining and particulate density. *Ex vivo* alveolar macrophages have undergone cytospin preparation, and staining with Fields B. Panels **a** and **b** show representative 40x light microscopy images of macrophages from low and high particulate groups respectively

Forty-four participants were selected for 16S RNA sequencing (23 from low particulate and 21 from the high particulate group as determined by alveolar macrophage carbon content from an available set of 128 samples (representative images are shown in Fig. 1). Baseline characteristics are given in Table 1. Participants in the low and high particulate groups did not differ significantly in terms of sex, BMI, lung function, source of fuels used for cooking or lighting, or bronchoalveolar lavage differential cell counts (see Table 1). High particulate individuals were older (mean 34.1 years vs. 29.2 years, *p* = 0.03). All individuals did have a potential domestic source of particulate exposure for either cooking or lighting.

**Table 1:** Characteristics of participants classified as low and high macrophage particulate burden

	Low particulate (*n* = 23)	High particulate (*n* = 21)	*p*
*Age, mean years (SD)*	29.2 (7.7)	34.1 (6.8)	0.03
*Sex, female (%)*	9 (39)	12 (57)	0.37
*Ethnicity – African, n (%)*	23 (100)	21 (100)	-
*BMI, mean (SD)*	22.4 (4.2)	22.4 (2.6)	0.97
*FEV1 % predicted, mean (SD)*	96.2 (15.2)	98.2 (9.7)	0.62
*FVC % predicted, mean (SD)*	96.3 (15.1)	100.4 (9.5)	0.31
*Cooking, n (%)*			
*Electricity only*	2 (8.7)	0 (0)	0.60
*Charcoal stove*	14 (60.9)	13 (61.9)	.
*Wood*	7 (30.4)	8 (38.1)	.
*Lighting, n (%)*			
*Electricity only*	6 (26.1)	4 (19.1)	0.11
*Candle mostly*	10 (43.5)	4 (19.1)	.
*Paraffin mostly*	7 (30.4)	13 (61.9)	.
*BAL differential count, % (SD)*			
*Macrophage*	95.5 (4.6)	95.3 (3.2)	0.85
*Lymphocyte*	4.0 (4.1)	4.2 (2.6)	0.87
*Macrophage carbon, % (SD)*	0.1 (0.1)	2.5 (1.8)	<0.0001

Data are presented as *n* (%) or mean ± SD. Significance testing used Fisher’s exact tests

*BMI* Body Mass Index, *SD* standard deviation

### Alpha and beta diversity

The mean total of high quality sequences was 7268, and was similar in low and high particulate groups (7928 [SD 4283] vs 6545 [SD 3183] respectively). There was no difference in alpha diversity metrics between low and high particulate groups by any measure at either genus or phylum level (Observed taxa number, Chao 1, ACE indices, Shannon, Simpson’s (1-D), and Pielou all *p* > 0.05).

Beta diversity was no different in low and high particulate groups for genus level (PERMANOVA *p* = 0.209). Analysis on phylum level shows no difference between low and high particulate groups (PERMANOVA *p* = 0.397). Interestingly, when dispersion of the two communities was analysed, the low particulate population tended to be more spread out compared to high particulate group, though this did not quite reach statistical significance (average distance to centroid: 0.544 for the low biomass group, 0.484 for the high biomass group, *p* = 0.096) at genus level.

### Microbial differences between low and high particulate groups

There were no significant differences between low and high particulate groups at the phylum level (data not shown).

Table 2 shows the relative abundance of the twenty genera represented at a frequency of 1 % or more of the total sequence reads. Neisseria abundance was significantly associated with the high particulate group, accounting for 4.98 % (SD 7.71) of total reads compared with 1.00 % (1.80) in the low particulate group (*p* = 0.01). This relationship was maintained after adjustment for age, sex, and cooking location (*p* = 0.046). Tropheryma was identified significantly less frequently in the high compared with low particulate group (0.97 % [2.99] vs 13.37 % [29.5] respectively, *p* = 0.046 unadjusted and *p* = 0.01 adjusted). Streptococcus was observed at higher relative abundance in the high particulate group (13.71 % [13.09] vs 6.77 % [7.28]): this was non-significant in the unadjusted analysis (*p* = 0.062), but significant (*p* = 0.045) after adjustment for potential confounding variables. Ralstonia appeared more abundant in high particulate groups, only before adjustment (*p* = 0.027, after adjustment *p* = 0.991).

**Table 2:** Genus level differences between low and high particulate groups

	Reads, % of total (SD)		*p* value	
	High	Low	Unadjusted	Adjusted
*Streptococcus*	13.71 (13.09)	6.77 (7.28)	0.062	0.045*
*Prevotella*	7.28 (7.95)	7.61 (11.00)	0.938	0.899
*Tropheryma*	0.97 (2.99)	13.37 (29.50)	0.046*	0.006*
*Paenibacillus*	4.68 (11.37)	3.04 (9.67)	0.702	0.355
*Corynebacterium*	5.43 (11.84)	2.22 (4.11)	0.127	0.498
*Petrobacter*	3.78 (8.83)	3.37 (8.08)	0.924	0.560
*Acidovorax*	3.12 (12.28)	4.02 (8.31)	0.825	0.198
*Neisseria*	4.98 (7.71)	1.00 (1.80)	0.010*	0.043*
*Propionibacterium*	2.86 (4.72)	2.03 (4.21)	0.592	0.430
*Veillonella*	2.50 (3.52)	2.28 (3.25)	0.907	0.763
*Sphingomonas*	1.68 (2.62)	2.37 (3.39)	0.591	0.909
*Ralstonia*	0.15 (0.40)	3.70 (17.05)	0.027*	0.991
*Bacillus*	1.67 (3.10)	1.80 (4.97)	0.917	0.619
*Akkermansia*	1.88 (2.85)	1.43 (1.71)	0.482	0.221
*Fusobacterium*	1.22 (2.39)	1.70 (2.96)	0.684	0.303
*Actinomyces*	1.41 (2.41)	1.20 (2.05)	0.825	0.980
*Porphyromonas*	1.24 (1.99)	1.27 (2.87)	0.984	0.784
*Gemella*	1.66 (2.05)	0.66 (0.81)	0.055	0.338
*Staphylococcus*	0.98 (1.98)	1.14 (2.32)	0.838	0.677
*Cloacibacterium*	0.79 (2.63)	1.21 (4.43)	0.775	0.129

The relative abundance of bacteria in the lung microbiome was compared by using negative binomial test. Raw and adjusted p values are given. Adjustments were made for differences in age, gender and cooking location, between high and low biomass groups

*denotes significant at *p* < 0.05. Rows are presented in descending order of relative abundance

### Petrobacter in Malawi

A comparative analysis of the lung microbiome in Malawian and US samples identified Petrobacter as commonly isolated amongst the Malawi participants (17/44). Given both the original and recent descriptions of this organism in oil reservoirs, we hypothesised that Petrobacter prevalence could be explained by exposure to domestic fuel combustion, in particular to paraffin for lighting [10, 11]. Therefore, we analysed individual factors which might associate with Petrobacter (see Table 3), particularly in relation to domestic biomass fuel exposure. For those participants, who used electricity exclusively for lighting, have significantly lower bacteria abundance (0.025 ± 0.080) compared to those people who use candle, no-glass paraffin lights, instead (4.61 ± 9.26, *p* = 0.00044). Cooking predominantly outside rather than indoors was associated with lower copy numbers (cooking outdoor 0.50 ± 1.20 vs. cooking indoor 5.53 ± 10.20, *p* = 0.014).

**Table 3:** Associations of Petrobacter abundance in the lung microbiome of healthy Malawians

	Petrobacter abundance (%)		*p*
	Yes	No	
*Sex: female*	6.32 ± 10.37	1.32 ± 5.53	0.40
*Cooking fuel: electricity only*	0 ± 0	3.74 ± 8.51	<0.001*
*Cooking location: mostly outdoor*	0.51 ± 1.20	5.53 ± 10.20	0.015*
*Lighting fuel: electricity only*	0.025 ± 0.080	4.61 ± 9.27	0.0004*
*“Ex-smoking” status*	4.36 ± 9.06	0 ± 0	0.79

GLM with negative binomial model shows associations of Petrobacter within the lung microbiome of Malawians, focussing on potential exposures to particulate exposures from domestic fuel use. The relative abundance of Petrobacter in each cohort was recorded as mean ± standard deviation

*significance at *p* < 0.05 level

## Discussion

Our study found that high particulate exposure as defined by alveolar macrophage carbon content was associated with altered relative abundance of bacteria within the lungs of healthy Malawian adults. Specifically, we report higher proportions of Neisseria and Streptococcus, and lower proportions of Tropheryma. However, lung microbiome diversity and the relative abundance at the phyla level were not significantly different based on our current sample size. Petrobacter was more abundant in individuals who used biomass fuels for cooking or lighting than in those who did not. Finally, particulate matter in alveolar macrophages did not directly correlate with biomass exposure as determined by questionnaire, suggesting in this small study that other factors contributed to alveolar macrophage particulate ingestion. These likely include ingress of pollution from neighbouring homes, traffic pollution when commuting to work and occupational exposures.

This study provides a novel analysis of the lung microbiome from individuals in a low income country with exposure to high biomass fuels. We characterised high particulate exposures according to the particulate burden in the cells of the distal airways, which reflects the cumulative exposure to respirable-size particulates. This is likely to be more relevant to changes in the microbiome than air sampling methods [23]. We did not find any significant differences in the lung microbiome *as a whole* between high and low particulate groups. However, both richness and abundance of the lower respiratory microbiome may be significantly altered by sampling techniques which vary in their potential for introducing “carryover” contamination from the upper airways [24]. Individual lower respiratory tract microbiome demonstrates less similarity to that in other individuals than to the upper respiratory tract of the same person [9, 25, 26]. Nevertheless, samples obtained directly from explanted lungs and those taken indirectly by bronchoscopy demonstrate similar patterns [24]. Our study did not include sampling of the upper airway. Therefore, one cannot determine conclusively whether our BAL findings reflect true differences in the lung microbiome or differences in upper airway carriage between subjects with low and high exposure to particulates. However, since micro-aspiration is common in humans [17], it is likely that a true lung microbiome will contain many of the same taxa found in the oral cavity. Our study was performed on stored samples: not all of the potential environmental controls were available. However, since all subjects underwent the same bronchoscopy protocol, findings between high and low biomass fuel groups are highly likely to represent non-artefactual differences. Sterile saline controls from the bronchoscope using the same kit as this study have been reported, and contain a very low number of reads [27].

Reduced diversity of the lung microbiome has been seen in disease states, such as COPD, [24] and with medical treatments, such as inhaled corticosteroids [28]. In our HIV-negative, healthy volunteers, however, we found no significant differences in diversity or richness between high and low particulate groups, mirroring findings of comparisons between smokers and non-smokers in the US [9]. Our study is compatible with prior descriptions of high diversity in the lung microbiome, with Streptococcus and Prevotella most frequently represented [9, 24].

Despite the absence of differences in the global lung microbiome between high and low particulate exposed subjects, interesting differences in specific taxa were observed. Streptococcus was more abundant in high particulate exposed participants after adjustment of potential confounders. In humans, ambient air pollution increases the risk of pneumonia in adults and children, [29, 30] and is commonly caused by *Streptococcus pneumoniae*. Analagous changes occur in cigarette smokers [31]. Bronchial epithelium exposed to urban particulates demonstrates increase expression of platelet activating factor and *S. pneumoniae* binding [32]. Nasopharyngeal carriage of *S. pneumoniae* is high in Malawi, occurs early, and pathogen specific mucosal T-cell regulation may contribute to prolonged carriage [33]. High levels of Streptococcus have also been demonstrated in the upper airway microbiome in a case-control study of infants in Ecuador [34]. Similarities with the Malawian study are low levels of pneumococcal immunisation and antibiotic use, and significant rural poverty. Taken together, these studies would provide a mechanistic link between our findings and the epidemiological associations of particulate concentration and pneumonia incidence.

Neisseria was also more prominent in high particulate group. This organism is not usually considered in the context of the lower airways, although serogroup Y has been associated with pneumonia in the elderly and Army recruits [35]. Nasopharyngeal carriage of *N. meningitidis* is increased amongst those exposed to cigarette smoke, [36] and *ex vivo* human epithelial cell models demonstrate bacterial binding is also increased [37]. The role of other ambient particulates is not well defined.

Tropheryma showed the inverse relationship, in that it was more frequently represented in the low particulate group. The pathogenic significance of this is uncertain: while *T. whipplei* is the aetiological agent of Whipple’s disease, it is commonly found in health in other studies of lung microbiome [9]. It occurs in especially high levels in microbiome studies of HIV-infected individuals from the US [38]. However, the lower levels in our particulate exposed healthy volunteers is unexplained.

While we hypothesised that particulate exposure could alter the microbiome, it is also possible that difference in the microbiome could affect particulate uptake in macrophages through effects on immune activation and clearance responses [39].

### Petrobacter and fuel use

The finding of Petrobacter, an unusual organism associated with fossil fuels, in lung lavage led us to speculate that biomass fuel use could be the source of this bacterium. Our analysis demonstrates the presence of Petrobacter is negatively associated with use of clean fuel (electricity) for cooking and lighting, and with practices that reduce household air pollution exposure (i.e. cooking outside). This, and the tendency to be increased in females, who are most highly exposed to HAP, [2] suggests that Petrobacter is associated with burning of biomass fuel in Malawians. Only recognised as a genus in 2004, Petrobacter are non-spore forming gram negative aerobic rods with flagella [10]. First isolated from an Australian terrestrial oil reservoir, the organism appears tolerant of high temperatures, and there are no relevant reports of human disease or pathological association. Overall, it is plausible, and supported by our study, that Petrobacter in the lungs derives from biomass fuels. Interestingly, there was no difference in the amount of Petrobacter in BAL from individuals with high and low particulate matter in alveolar macrophages. This may suggest that other sources of lower airway particulate matter are more important than using oil for cooking (i.e. use of other fossil fuels for cooking and heating, tobacco smoking).

## Conclusions

There are significant differences in the composition of the lung microbiome of Malawians with differing levels of particulate exposure as determined by macrophage carbon content. These differences might contribute to the excess respiratory infections associated with particulate exposure. We have further demonstrated that the finding of Petrobacter in the lung is associated with household biomass fuel exposure. Interventions to improve air quality have the potential to alter the microbiome of the lower respiratory tract and ultimately improve the lung health of Malawians.
